# Diagnosis and Management of a Patient with Congenitally Missing Maxillary First Permanent Molars: A Rare Case Report

**DOI:** 10.1155/2016/5891705

**Published:** 2016-07-25

**Authors:** Megha Gupta, Suman Panda, Fahad Ahmed Mutawwam, Fahad Musawi Mohammed Kariri

**Affiliations:** ^1^Department of Preventive Dental Sciences, College of Dentistry, Jazan University, P.O. Box 114, Gizan 45142, Saudi Arabia; ^2^College of Dentistry, Jazan University, P.O. Box 114, Gizan 45142, Saudi Arabia

## Abstract

Congenitally missing teeth are the most commonly seen dental anomalies. Agenesis of the permanent first molar has the least frequency of all the tooth types, and it usually occurs in association with oligodontia or anodontia. Thus, agenesis of the bilateral maxillary first permanent molar is an extremely rare occurrence, and no such case has been reported in ethnic Saudi Arabian population. We hereby report a case of nonsyndromic bilateral congenitally missing maxillary first permanent molar in an eight-year-old Saudi female patient. Comprehensive oral rehabilitation was done for the patient. The implications of the tooth agenesis are also discussed. The prognosis of this case is presented.

## 1. Introduction

 Hypodontia is the term used to describe the developmental absence of one or more primary or permanent teeth, excluding the third molars. It is the most commonly seen dental anomaly and is found more in the permanent dentition [1]. A tooth is diagnosed as congenitally missing when it could not be identified or discerned radiographically on the basis of calcification and there was no evidence of extraction [2].

The occurrence of a congenitally missing first permanent molar is a rare finding as the common congenitally missing permanent teeth in the order of frequency are third molars, second premolars, and lateral incisors [3]. Congenitally missing teeth are common in patients with cleft lip and palate, ectodermal dysplasia, Down syndrome, and so forth [4]; however, it is a rare finding in a nonsyndromic healthy patient without the absence of the other teeth.

Several hypotheses [5, 6] have been proposed to explain the etiology of the congenitally missing teeth like Butler's field theory, Svinhufvud's anatomic model, and Kjaer's neuroosteological developmental fields in the jaws. All these theories concluded that the permanent first molars are the most stable teeth in the jaw and have the least likelihood of being absent, except in those patients whose complete molar tooth series are absent, as seen in severe oligodontia or anodontia.

## 2. Case Report

An eight-year-old female patient reported to the Department of Pediatric Dentistry, College of Dentistry, Gizan, for routine dental examination. This was the patient's first dental visit. The patient was moderately built and nourished and the medical history was noncontributory. Extraorally, she exhibited a straight profile and normal facial appearance. The lips were competent.

**Figure 1:**
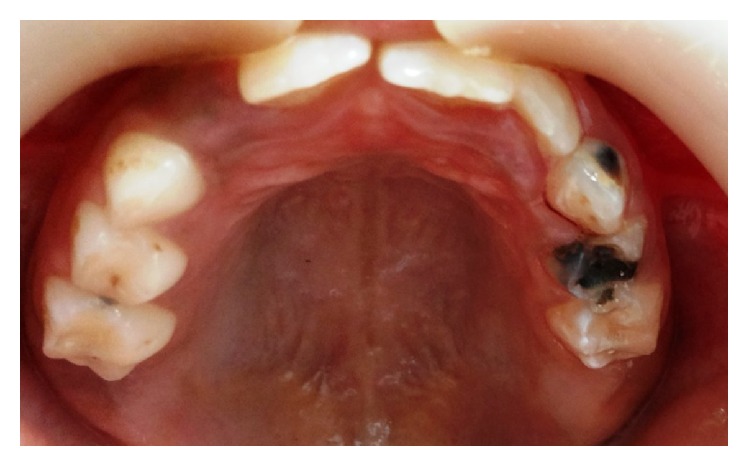
Intraoral picture of the maxillary arch at the age of eight years.

**Figure 2:**
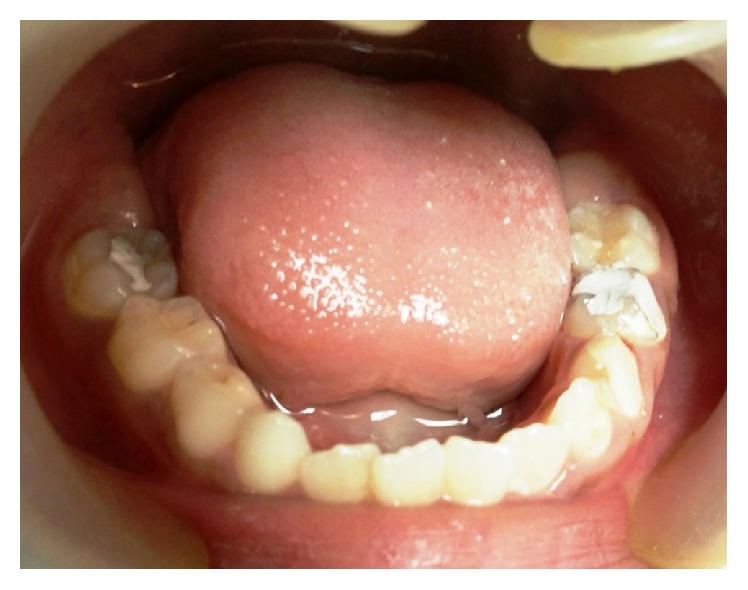
Intraoral picture of the mandibular arch at the age of eight years.

Intraoral examination revealed carious left primary maxillary canine and molars (63, 64, and 65), primary mandibular first and second molars (74 and 75), and right and left mandibular first permanent molars (36 and 46). Another remarkable finding was the bilaterally missing maxillary first permanent molars (16 and 26) (Figure 1). There was no history of previous extraction and this was the patient's first dental visit. However, in the mandibular arch, all the teeth according to the patient's chronological age were clinically evident (Figure 2). So, the following teeth were present intraorally:

**Table:** 


555453112122636465468584834241313273747536

**Figure 3:**
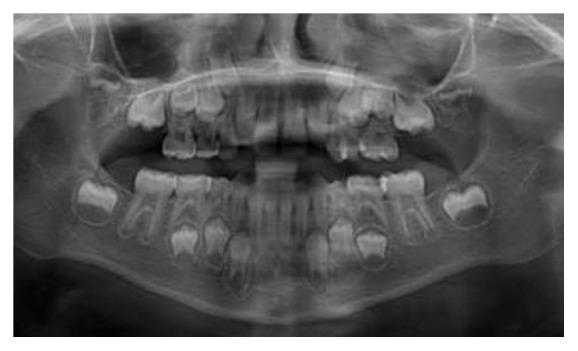
Panoramic X-ray photograph at the age of eight years showing agenesis of 16 and 26.

An orthopantomograph was then taken. It confirmed our clinical finding and revealed the agenesis of the maxillary first molars (16 and 26) (Figure 3). On the contrary, the mandibular arch showed the presence of all teeth (except the tooth buds of the third molar). The tooth formation of the permanent second molars corresponded well in the maxillary and the mandibular arch. The third molar tooth germs were evident in the maxillary arch but were absent in the mandibular arch. The primary molar relation was mesial step. The patient's parents were dentate and reported no missing teeth or associated syndromes among their other family members. The siblings of the patient that included a brother and a sister exhibited normal complement of the dentition.

**Figure 4:**
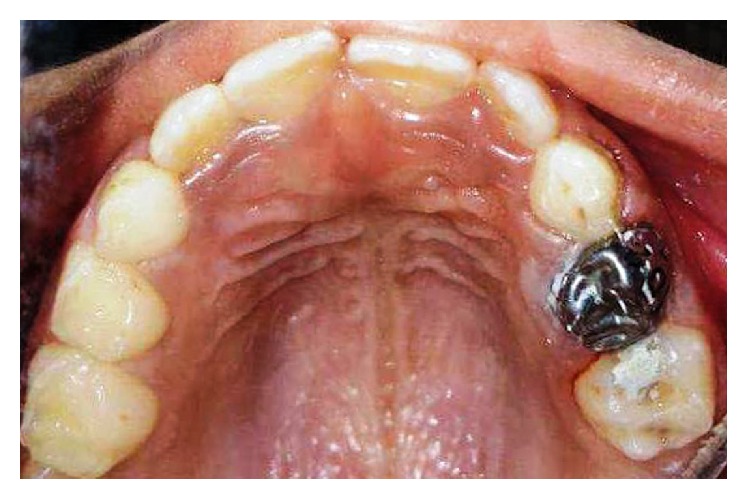
Intraoral picture of the maxillary arch after one year.

**Figure 5:**
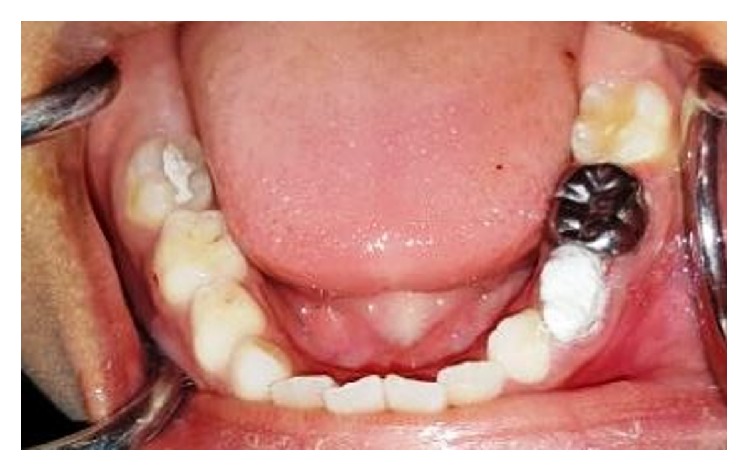
Intraoral picture of the mandibular arch after one year.

The parents were informed of the missing teeth. Glass ionomer restoration was done for all the carious molars. Stainless steel crown was placed over the multisurface restoration done on the left maxillary primary first molar (64) and the right mandibular primary second molar (75). Enamel defects on the primary maxillary right canine and primary mandibular left canine (53 and 73) were restored with composite resin. The patient is currently under follow-up every six months to monitor the eruption of the permanent maxillary second molar (Figures 4 and 5). Written informed consent was obtained from the patient's father regarding the use of the patient's data for scientific and teaching purpose.

## 3. Discussion 

The permanent first molars are considered as the most important teeth in the dentition. They are the largest and the strongest teeth, because of their bulk and anchorage. They perform the important function of mastication of food. Angle's classification of the malocclusion is also based on the relationship of the permanent first molars when in occlusion. Hence, the absence of the permanent first molar can have an adverse effect on the normal growth and development, phonetics, and occlusion and cause orthodontic problems [7]. Unlike the other permanent teeth, the permanent first molars do not have any preceding primary teeth. Their enamel organ arises directly from the dental lamina. They emerge into the oral cavity at an average age of 5.5 to 7 years, distal to the primary second molars [8].

Congenitally missing permanent first molars fall under the rarest of the rare cases in normal, healthy individuals. Agenesis of the permanent maxillary first molars does not occur frequently in the general population. A recent meta-analysis reported the prevalence rates from 0.02 to 0.05% and 0 to 0.02% for the maxillary and mandibular missing permanent first molars [9].

In our case, the maxillary permanent first permanent molars are likely to be congenitally missing because the dental age of the tooth germ in the maxillary molar region (Nolla's stage 6, i.e., crown completely formed) [10] does not coincide with that of the mandibular permanent first molars 36 and 46 (Nolla's stage 9, i.e., root completely formed but with open apex) at chronological age range of 8-9. The tooth formation stage of the maxillary permanent molar corresponds well with that of the mandibular second molar. Further, the position of the tooth germs also suggests that they are permanent second molars, rather than delayed eruption of first molars, as they would have mesially migrated in the absence of the first permanent molar tooth bud. Further, the eruption time of the permanent first molars into the oral cavity is considered to be the least variable among all permanent teeth [11].

The deft index of the patient was recorded as 6, DMFT as 2. All the treated teeth were diagnosed as reversible pulpitis. The permanent mandibular molars were in good health. This clearly rules out any chances of early extraction of permanent maxillary first molar due to caries; and the same was elicited from the patient's past dental history also.

Hence, all these findings support the hypothesis that the maxillary permanent molars present in our case are certainly the second molars associated with the congenital absence of the maxillary first molars. A PubMed literature search revealed that no such case has been reported in the ethnic Saudi Arabian population.

Despite the low prevalence rate, maxillary first permanent molar agenesis presents clinically significant problems affecting treatment planning and outcome, because the first molars play an important role in the mastication of food, in supporting the vertical dimension of the face, and as anchorage teeth against orthodontic forces. The type of malocclusion, severity of crowding, and facial profile are of major concern in determining the final treatment plan [12]. In our case, in the absence of the permanent first molar, the maxillary permanent second molars may freely migrate into a favorable position, rather than tipping, due to the lower resistance of maxillary trabecular alveolar bone in comparison to that of the mandible.

## 4. Conclusion 

Congenitally missing permanent first molars are a rare entity. Permanent first molars have very strategic importance in the dentition because of their function and stability. Routine radiographic examination is mandatory for patients in whom certain teeth do not erupt at their scheduled time. It is important to recognize the anomaly as early as possible, to guide the occlusion, and to implement multidisciplinary management.
